# Using a co-design approach to develop, implement, and evaluate a Preventative Online Mental Health Program for Youth (POMHPY)

**DOI:** 10.1192/j.eurpsy.2025.697

**Published:** 2025-08-26

**Authors:** S. Kim, E. Moghimi

**Affiliations:** 1 Psychiatry and Behavioural Neurosciences, McMaster University, Hamilton; 2 Waypoint Research Institute, Waypoint Centre for Mental Health Care, Penetanguishene; 3 Psychiatry, Queen’s University, Kingston, Canada

## Abstract

**Introduction:**

From March 2020 to 2021, the risk of youth developing a mental health issue increased by 50% in Canada. To address the detrimental effects of the COVID-19 pandemic, this project collaborated with youth and community partners in Ontario, Canada, to co-design a Preventative Online Mental Health Program for Youth (POMHPY) focused on improving mental, physical, and social well-being.

**Objectives:**

1. To co-design a preventative online mental health program tailored to the needs of Ontario youth. (2) To evaluate the program’s efficacy in improving mental well-being and health-related quality of life. (3) To engage youth in the development and continuous improvement of the program.

**Methods:**

Initially, literature reviews were conducted to identify evidence-based programs that could be integrated into POMHPY. Surveys and focus groups were used to capture youths’ mental health concerns and program needs. The findings were presented to community partners for additional feedback and refinement of the program. A second survey and focus group explored the likelihood of program use and piloted the first session. Subsequently, 53 youths (mean age=19.15) participated in the POMHPY program during the summer of 2023. Pre-, post-, and follow-up surveys measuring mental well-being were administered. Preliminary descriptive statistics and t-test analysis were conducted to measure the program’s efficacy. A subset of participants (n = 21) attended 90-minute focus groups to discuss program perceptions, perceived benefits, impact on personal life, and areas of improvement.

**Results:**

Youths’ mental well-being, measured by the Warwick-Edinburgh Mental Well-being Scale, significantly improved after the completion of the program [t (24) =-2.91, p=.008]. Health-related quality of life, measured by the AqoL-6D, also significantly improved [t (6) =-3.34, p=.016]. These improvements were maintained one month after completing the program. Participants viewed the skills and strategies learned in POMHPY as beneficial in improving their stress and well-being. Peer facilitators in the same age range as participants contributed to meaningful discussions and interactive activities that contrasted with a lecture-style learning environment. Suggestions for improvement included flexible scheduling, increasing reminders, and enhancing understanding of program components.

**Conclusions:**

Preliminary analysis supports the program’s efficacy in improving mental well-being and health-related quality of life. Participants also reported a positive experience with the program and suggested improvements for integration. The program will be scaled nationally in the next phase, ensuring broader access to preventative mental health care for youth across Canada.

**Disclosure of Interest:**

None Declared

